# A mixed methods study of the teachers’ self-efficacy views and their ability to improve self-efficacy beliefs during teaching

**DOI:** 10.3389/fpsyg.2022.1035829

**Published:** 2023-01-04

**Authors:** Şenol Orakcı, Derya Yüreğilli Göksu, Savaş Karagöz

**Affiliations:** ^1^Department of Educational Sciences, Aksaray Faculty of Education, Aksaray University, Aksaray, Türkiye; ^2^Ministry of National Education, Etimesgut BİLSEM, Ankara, Türkiye; ^3^Department of Educational Sciences, Aksaray Faculty of Education, Aksaray University, Aksaray, Türkiye

**Keywords:** self-efficacy, teaching, teacher, mixed method, Turkey

## Abstract

**Objective:**

The current study has the aim of investigating teachers’ views about their self-efficacy and how they improve their self-efficacy beliefs during teaching practice.

**Methods:**

The study was designed in a mixed methods research design. “Teacher’s Self-Efficacy Scale,” “Personal Information Form” and “Semi-structured Interview Form” were employed in the study. The quantitative data were collected from 379 teachers in public schools in the 2021–2022 academic year, whereas the qualitative data were obtained from the top 10 participants with the highest level of self-efficacy.

**Results and Discussion:**

Based on the qualitative and quantitative results of the present study, it was revealed that teachers’ self-efficacy levels were high, and they felt self-efficient in their teaching.The study is of great importance since determining teachers’ opinions about their self-efficacy beliefs and how they improve their self-efficacy beliefs in the solution of the problems they encountered during teaching practice will not only raise awareness of the importance of self-efficacy in teaching profession, but will contribute to further research and qualified teacher training.

## Introduction

Unlike many other professions, the cognitive, affective and psychomotor characteristics of teachers in the teaching profession affect the quality and amount of knowledge, skills, values, and attitudes that students will acquire. One of the factors affecting the performance in the teaching profession is the extent to which teachers perceive themselves to be self-efficient in carrying out their teaching profession, which can be expressed as the perception of teaching self-efficacy. Numerous studies have been conducted on teacher self-efficacy which has been the subject of extensive research over the past 30 years (Klassen and Chiu, 2010; Klassen and Chiu, 2011; Klassen et al., 2011; Klassen and Tze, 2014; Chesnut and Burley, 2015; Zee and Koomen, 2016; Gale et al., 2021). In fact, teachers’ self-efficacy has gradually taken on a more significant role in school psychology research due to its consequences for instructional practices, teaching effectiveness, and student academic achievement. Teachers’ self-efficacy, or their confidence in their ability to successfully manage the responsibilities, demands, and problems associated with their professional activity, has a significant impact on teaching profession because effective teachers appear to possess a strong sense of efficacy. It is clear that teachers’ self-efficacy is highly effective and significantly influences their pedagogical growth in a variety of ways (Barni et al., 2019; Alibakhshi et al., 2020). In this context, more clarification of self-efficacy belief is considered extremely significant. Self-efficacy that has a vital role in the competencies of individuals is related to the ability of individuals to do their jobs properly and their beliefs to be successful. “Self-efficacy” theory deals with the diversity of individuals and abilities. Although individuals have similar abilities, their performance in very different and even extraordinary conditions depends on their individual beliefs (Bandura, 1986). Bandura (1997) defined self-confidence conceptually as a “belief in one’s capabilities to organize and execute the courses of action required to attain goals” (p. 3). Self-efficacy is a person’s perception or evaluation in their ability to succeed in a particular situation.

The belief in one’s own ability to carry out a task is also known as self-efficacy, which represents the state of people viewing themselves as competent in a subject. One’s performance and motivation are both positively impacted by this belief. It is believed that a person’s self-efficacy belief will have a favorable impact on their capacity to deal with issues and develop new tactics. The objectives that people set for themselves, the amount of work they put in and how long they can overcome with the difficulties to reach their goals, as well as how they react when they fail, are all influenced by their self-efficacy beliefs (Klassen and Chiu, 2011; Klassen and Tze, 2014; Zee and Koomen, 2016; Gale et al., 2021).

In addition, “How teachers see themselves in terms of fulfilling the requirements of the teaching profession” can be explained with the concept of teacher self-efficacy (Gibson and Dembo, 1984; Atıcı, 2002; Schunk, 2014). Teacher self-efficacy is a very important concept in terms of instruction process. Teachers with high levels of self-efficacy are more committed to their work (Glickman and Tamashiro, 1982; Coladarci, 1992) and positively affect students’ achievement levels. In addition, teachers with high level of self-efficacy effectively manage their classroom and time and prevent undesirable student behaviors as well as applying new teaching methods (Woolfolk and Hoy, 1990). Additionally, teachers with high level of self-efficacy spend more time and effort on their students, treat them more ethically, take more responsibility, provide a positive classroom environment, and are inclined to identify students’ needs. They also help students with learning difficulties, try new ways and give advice to them to be successful, which contributes their students’ academic performance and positively affect students’ achievement levels (e.g., Gibson and Dembo, 1984; Midgley et al., 1989; Guskey and Passaro, 1994; Woolfolk-Hoy and Burke-Spero, 2005; Caprara et al., 2006; Shidler, 2009; Elliott et al., 2010; Guo et al., 2010; Tschannen-Moran and Johnson, 2011; Marzano, 2017). However, teachers who have low levels of self-efficacy spend more time on non-academic subjects, criticize learners in case of failure, make less effort to find materials, apply more teacher-centered methods, and avoid activities that they think will exceed their capacity (Bandura, 1995; Schunk and Pajares, 2009; Swackhamer et al., 2009).

In short, self-efficacy belief has a vital role in helping the teacher, who is responsible for raising individuals who can keep up with the times as well as acquiring the necessary knowledge and skills and following the innovations (Koç, 2013). The fact that teacher candidates and teachers have professional competencies is related to their beliefs that they receive quality education. Self-efficacy is the most important among these beliefs (Kahyaoğlu, 2011). The teacher plays an important role in the process of acquiring the predetermined objectives with the teaching activities in schools. For this reason, a teacher’s professional self-efficacy belief must be high to create a positive learning environment (Akkoyunlu et al., 2005; Yokuş, 2014), and the teacher’s efficacy belief significantly affects their classroom practices as well as learning and teaching (e.g., Caprara et al., 2006; Elliott et al., 2010; Kahyaoğlu, 2011). The abilities and skills of teachers have a crucial role in contributing qualified teaching and overcoming the difficulties they encounter during teaching (Özdemir, 2008). There exists a correlation between teachers’ general culture, content knowledge, pedagogical knowledge and skills and self-efficacy (Yeşilyurt, 2013). In other words, it is not enough for a teacher to have only professional knowledge to practice his/her profession. In addition, the teacher’s self-belief in performing his/her profession is also important (Güneş et al., 2015). In this regard, it may be difficult for teachers who do not find themselves professionally equipped, competent as well as self-efficient to achieve professional success (Aydın et al., 2014).

In order for a qualified education to be realized, the teacher must have faith in himself/herself in terms of teaching competence that will increase the achievement level of the students. The teacher who has these beliefs takes the responsibility of students’ learning by using new teaching strategies and techniques (Kurbanoğlu, 2004). In this context, teachers’ self-efficacy beliefs can be stated to be efficient in their students’ success and motivation as well as problem solving and effective planning (Özdemir, 2008). In short, teacher self-efficacy reveals itself the self-confidence of him/her that he/she can overcome the obstacles that may arise in the way of students for the purpose of achieving the goals planned in the education process (Tabancalı and Çelik, 2013). In the meanwhile, the determinant of the teacher’s behavior in the classroom can be explained to be related to the teacher’s “self-efficacy” perceptions (Orakcı and Durnalı, 2022).

As shown above, there are a lot of studies investigating teachers’ self-efficacy beliefs, (e.g., Woolfolk-Hoy and Burke-Spero, 2005; Meristo and Eisenschmidt, 2014; Dicke et al., 2015; Walsh et al., 2020; Xiyun et al., 2022), but not much is known about how they improve their self-efficacy beliefs in the solution of the problems during teaching in Turkey, a centralized country, which is a “gap” in the related literature. In addition, according to Tschannen-Moran and Woolfolk Hoy (2001), a teacher’s self-efficacy belief is influenced by their perception of their own feelings and knowledge as well as the potential impact of culture and society on the roles, social interactions, and expectations of teachers. Since it reflects the fundamental belief systems of teachers, investigating teachers’ self-efficacy can be very beneficial in the quest to find ways to increase teachers’ effectiveness in teaching, which increases the importance of the study. As it is seen in the literature, the concept of “self-efficacy” is frequently discussed in educational research and is seen as one of the important elements of affective characteristics. Self-efficacy perceptions of teachers are important in increasing their professional success and productivity (Pajares, 1996). The most important effect in creating a qualified learning-teaching environment is the teacher’s perception of self-efficacy (Lortie, 1975; Ashton, 1984; Woolfolk and Hoy, 1990; Tobin et al., 1994; Tschannen-Moran and Woolfolk Hoy, 2001). According to Tschannen-Moran and Woolfolk Hoy (2002), it is advocated that the higher the teachers’ perception of self-efficacy is, the more effective the success, motivation and development of self-efficacy perceptions of the students to be trained by them will be. In this respect, determining teachers’ opinions about their self-efficacy beliefs and how they improve their self-efficacy beliefs in the solution of the problems they encountered during teaching practice will not only raise awareness of the importance of self-efficacy in teaching profession, but also will contribute to further research and qualified teacher training.

Based on the purpose of filling the above-mentioned gap in the literature, our research attempts to address the following questions:

1. What are the teachers’ level of self-efficacy?
2. Do teachers’ self-efficacy differ significantly with regard to their gender, seniority and education level?
3. What are the teachers’ views on their own self-efficacy beliefs?

## Research method

**Figure 1:**
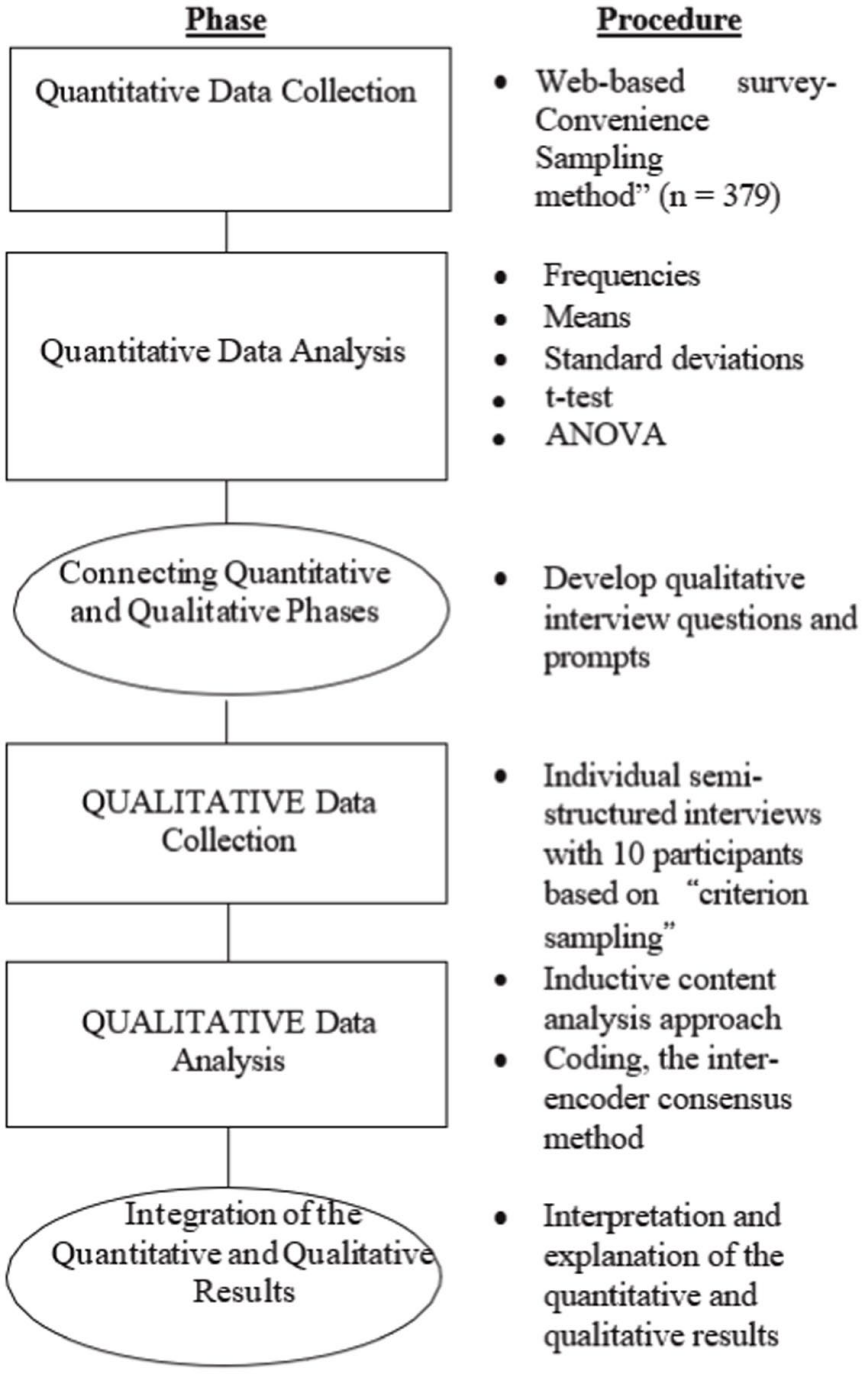
Mixed research: Explanatory sequential research design in the present study.

The present study used a mixed methods research design. The purpose of the mixed methods design in the context of the current study is to produce a more thorough and detailed picture of the participants’ self-efficacy. The study utilized an “explanatory sequential research design” by Creswell and Plano Clark (2011). In this design, the researcher initially collected quantitative data in order to attain a general response to the research problems, and then tried to deepen and elaborate on this general picture by means of the qualitative data that were collected in the second phase of the study (Creswell, 2013). The researchers preferred a mixed method approach because any adjustments that are made should be evaluated using mixed-methods approaches since self-efficacy is a complicated construct. Additionally, qualitative data can also be used to help interpret quantitative findings. Figure 1 illustrates the research methodology.

### Participants

Based on “convenience sampling method,” quantitative data were collected from 379 teachers in public schools in the 2021–2022 academic year. The data collection tools were delivered to participants through WhatsApp and Facebook Groups Links. Participation was voluntary. Demographic characteristics of the participants are presented in Table 1.

**Table 1:** Demographic characteristics of the participants.

Variables		*f*	Percent (%)
Gender	Male	188	49.6
	Female	191	50.4
Education level	Undergraduate	267	70.4
	Master	85	22.4
	PhD	27	7.1
Seniority	1–10 years	68	17.9
	11–20 years	125	32.9
	+21 years	186	49
School level	Primary School	89	23.5
	Secondary School	185	48.8
	High School	105	27.7

As Table 1 suggests, out of a total of 379 teachers (49.6% female, 50.4% male), 70.4% had undergraduate degree, 22.4% had master’s degree and 7.1% had doctoral degree. 49% of the participant teachers (*n* = 186) had +21 years previous experience of teaching, and the others had differing periods of teaching experience: 1–10 years (*n* = 68) and 11–20 years (*n* = 125). Finally, 23.5% of the participant teachers (*n* = 89) work at primary school, 48.8% (*n* = 185) work at secondary school and 27.7% (*n* = 105) at high school.

“Criterion sampling” was utilized to select the top 10 participants that met the criteria of having the highest level of self-efficacy in the “Teacher’s Self-Efficacy Scale” developed by Schmitz and Schwarzer (2000) and adapted into Turkish language by Yılmaz et al. (2004). In the scale, the participant teachers’ highest level of self-efficacy remarks constituted coping with difficult students, social interaction with them, teaching accomplishment, and skill development on teaching profession. Within this context, the top 10 participants with the highest level of self-efficacy were specifically identified. They were asked to participate voluntarily in the study. All of them agreed to participate in the interview. They were informed about confidentiality, anonymity and voluntary nature of participation based on a written informed consent form and the researchers explained them their right to take part in or leave from the research. Six of the participants out of 10 are female and 4 are male teachers. The age range of the participant teachers was 35–53. Their professional seniority ranges from 15 to 30 years. Of the teachers, three were primary school teachers, three English teachers, two maths teachers, and two Social Science teachers. Four of these teachers held a doctoral degree, four of them a masters’ degree, and two of them a bachelor’s degree. Interviewees were given a code that included the word “teacher” and a corresponding number in order to maintain participant confidentiality.

Necessary ethical permission dated 09.11.2021 and numbered 19632675 was obtained from the Ministry of National Education. Based on volunteerism, the teachers were asked if they would like to be involved in the study by providing a brief information about the research. After asking them to complete a written informed consent form, the researcher involved the teachers who would consent to participate in the study by giving them information about appropriate time and place for the interview.

### Data collection tools

“Teacher’s Self-Efficacy Scale,” “Personal Information Form” and “Semi-structured Interview Form” were employed in the study.

#### Teacher’s self-efficacy scale

The “Teacher’s Self-Efficacy Scale” developed by Schmitz and Schwarzer (2000) and adapted into Turkish language by Yılmaz et al. (2004) consists of one dimension and eight items while the number is 10 in original scale. As part of the current study, the “coefficients of concordance” computed based on the “Confirmatory Factor Analysis” “*χ*^2^/sd = 2.75”; “CFI = 0.95”; “NFI = 0.93”; “GFI = 0.91”; “AGFI = 0.94”; “RMSEA = 0.09”) were within the acceptable range and the overall “Cronbach’s Alpha coefficient” for the scale was computed as 0.90.

#### Semi-structured interview form

The data in the qualitative dimension of the study were collected through a semi-structured interview form prepared by the researcher. In this direction, a semi-structured interview form focusing on the experiences of participant teachers about self-efficacy was prepared in the study. In the interview form, there were six questions based on teachers’ self-efficacy beliefs. Two qualitative research experts evaluated them in terms of language, meaning, clarity and relevance to the subject. In line with the suggestions from the experts, some adjustments were made to make the questions more understandable and the interview form was made ready for application.

### Data analysis

In analysis of the quantitative data, frequencies, percentages, means, and standard deviations firstly were computed, and then “*t*-test” and “One-Way Variance Analysis (ANOVA)” were performed. As for the qualitative data analysis, a total of 283 min of interviews based on a total of 10 interviewees, each of whom the interviews lasted around 20 min were initially transcribed, and then the transcribed data were reviewed for accuracy and finalized. The research data were analyzed with an “inductive content analysis approach” (Patton, 2002). In the “inductive content analysis approach,” themes and categories are based on the data set (Zhang and Wildemuth, 2009). In other words, codes emerge from the expressions of the participants and form sets of meanings. This approach contributes to understanding the behavior of individuals and their nature. Similar data are handled by bringing concepts together and documents related to data are analyzed in a systematic way. In the context of this research, first of all, each data set was read in detail and the words/word groups serving the purpose of the research were determined and codes were created. After the coding was completed, the similarities and differences between the codes obtained were examined (Yıldırım and Şimşek, 2013). Themes were created by bringing together the codes that were related to each other. In the last phase, the themes were revised and checked by use of the inter-encoder consensus centered on the use of two qualitative research encoders. According to the reliability formula developed by Miles and Huberman in 1994, [*P* = (number of agreements/total number of agreements + disagreements) 100], an agreement of 81% was attained, which is regarded as reliable for study (Miles and Huberman, 1994).

## Findings

### Quantitative findings

The quantitative findings below are firstly given in the order of the sub-problems of the study.

## What are the teachers’ levels of self-efficacy?

Table 2 presents a descriptive analysis of teachers’ self-efficacy levels.

**Table 2:** Descriptive statistics for participants’ self-efficacy levels.

	Item	x	S.D
1	I know that I can maintain a positive relationship with parents even when tensions arise.	3.27	1.11
2	When I try really hard, I am able to reach even the most difficult students	4.75	0.49
3	I am convinced that, as time goes by, I will continue to become more and more capable of helping to address my students’ needs.	3.27	1.11
4	Even if I get disrupted while teaching, I am confident that I can maintain my composure and continue to teach well	3.87	1.03
5	I am confident in my ability to be responsive to my students’ needs even if I am having a bad day.	4.50	0.63
6	I am convinced that I can develop creative ways to cope with system constraints (such as budget cuts and other administrative problems) and continue to teach well.	3.94	0.90
7	I know that I can motivate my students to participate in innovative projects.	4.31	0.79
8	I know that I can carry out innovative projects even when I am opposed by skeptical colleagues.	4.19	0.83

Among the responses by the teachers to the “Teacher Self-Efficacy Scale” (TSES), the highest mean scores belonged to the remarks such as “When I try really hard, I am able to reach even the most difficult students,” “I am confident in my ability to be responsive to my students’ needs even if I am having a bad day,” and “I know that I can motivate my students to participate in innovative projects.”

On the other hand, the lowest mean scores referred to the remarks such as “I know that I can maintain a positive relationship with parents even when tensions arise.,” “I am convinced that, as time goes by, I will continue to become more and more capable of helping to address my students’ needs,” and “Even if I get disrupted while teaching, I am confident that I can maintain my composure and continue to teach well.”

Overall, teachers’ self-efficacy levels are high, and they feel self-efficient in their teaching. Additionally, it can be inferred from the participant teachers’ remarks that coping with difficult students, social interaction with them, teaching accomplishment, and skill development on teaching profession are main domains for which the participants may have varying expectations about their own self-efficacy. In fact, it can be said that these crucial domains seem to be crucial for effective education.

## Do teachers’ self-efficacy differ significantly in regard to their gender, education level, seniority and the school level?

Table 3 presents “*t*-test” results to analyze “Teacher Self-Efficacy Scale” (TSES) for gender.

**Table 3:** “*T*-test” results to analyze “TSES” for gender.

	Gender	*n*	*x-*	SD	df	*T*	*P*
TSES	Female	188	42.69	8.70	377	1.592	0.113
	Male	191	41.87	11.17			

*p* < 0.05.

As in Table 3, “self-efficacy” (*t* = 1.592, *p* > 0.05) scores were not found to differ significantly by gender. It can be inferred from the present finding that gender did not make any significant difference regarding the participant teachers’ self-efficacy beliefs and that both male and female participants held similar views about their own self-efficacy beliefs.

Table 4 below presents “ANOVA test” results for “TSES” and education level.

**Table 4:** “ANOVA test” results for “TSES” in regard to education level.

Scale	Group	*N*	Mean	SD	Source of variation	Sum of squares	df	Mean square	*F*	*p*	Significant difference^*^
TSES	1. Undergraduate	267	29.14	5.16	Between Groups	2012,887	2	1086.979	18.673	0.000	3–1
	2. Master	85	37.67	7.63	Within groups	39,515,987	376	66.790			
	3. PhD	27	41.88	7.10	Total	37,689,981	378				3–2

^*^Parametric Dunnet multiple comparison test was conducted to analyze whether or not there was a significant difference between group or groups.

As seen in Table 4, the study also uncovered a significant difference between scores of “teachers’ self-efficacy” in regard to education level (*F* = 18.673; *p* < 0.05), which reveals that those with PhD ( $\bar{X}$ = 41.88) had significantly higher “self-efficacy” scores than those with master ( $\bar{X}$ = 37.67) and those with undergraduate ( $\bar{X}$ = 29.14). It can be concluded that the participant teachers’ education level created a significant difference in their self-efficacy beliefs, which supports that their higher level of education affects their self-efficacy” beliefs more positively.

Table 5 presents “ANOVA test” results for “TSES” and seniority.

**Table 5:** “ANOVA test” results for “TSES” in regard to seniority.

Scale	Group	*N*	Mean	SD	Source of variation	Sum of squares	df	Mean square	*F*	*p*	Significant difference^*^
TSES	1. 1–10 years	68	41.82	15.13	Between Groups	5535.593	5	1387.96	4.589	0.000	1–2
	2. 11–20 years	125	45.29	16.24	Within groups	77892.287	373	336.889			1–3
	3. +21 years	186	49.93	14.19	Total	83427.880	378				2–3

^*^Parametric Dunnet multiple comparison test” was conducted to analyze whether or not there was a significant difference between group or groups.

As seen in Table 5, the study uncovered a significant difference (*F* = 4.589; *p* < 0.05) between scores of “self-efficacy” in regard to seniority, which shows that teachers with greater seniority (+21 years; $\bar{X}$ = 49.93) had higher “self-efficacy” scores than teachers with 11–20 years ( $\bar{X}$ = 45.29) and teachers with 1–10 years ( $\bar{X}$ = 41.82). In addition, teachers with 11–20 years ( $\bar{X}$ = 45.29) had higher “self-efficacy” scores than teachers with less seniority (1–10 years; $\bar{X}$ = 41.82). It can be inferred that teachers’ seniority made a significant difference in their self-efficacy beliefs, which supports that their higher seniority affects their self-efficacy” beliefs more positively.

Table 6 presents “ANOVA test” results for “TSES” and the school level.

**Table 6:** “ANOVA test” results for “TSES” in regard to the school level.

	Group	*N*	Mean	SD	*F*	*p*
TSES scale	1. Primary school	89	28.19	5.23	2.012	0.116
	2. Secondary school	185	27.68	6.66		
	3. High school	105	29.45	6.19		

*p* > 0.05.

Table 6 shows that there is no statistically significant difference between total scores of teachers’ self-efficacy perceptions in terms of the school level (*p* > 0.05). In other words, it might be suggested that teachers’ self-efficacy perceptions are similar in terms of the school level.

### Qualitative findings

**Figure 2:**
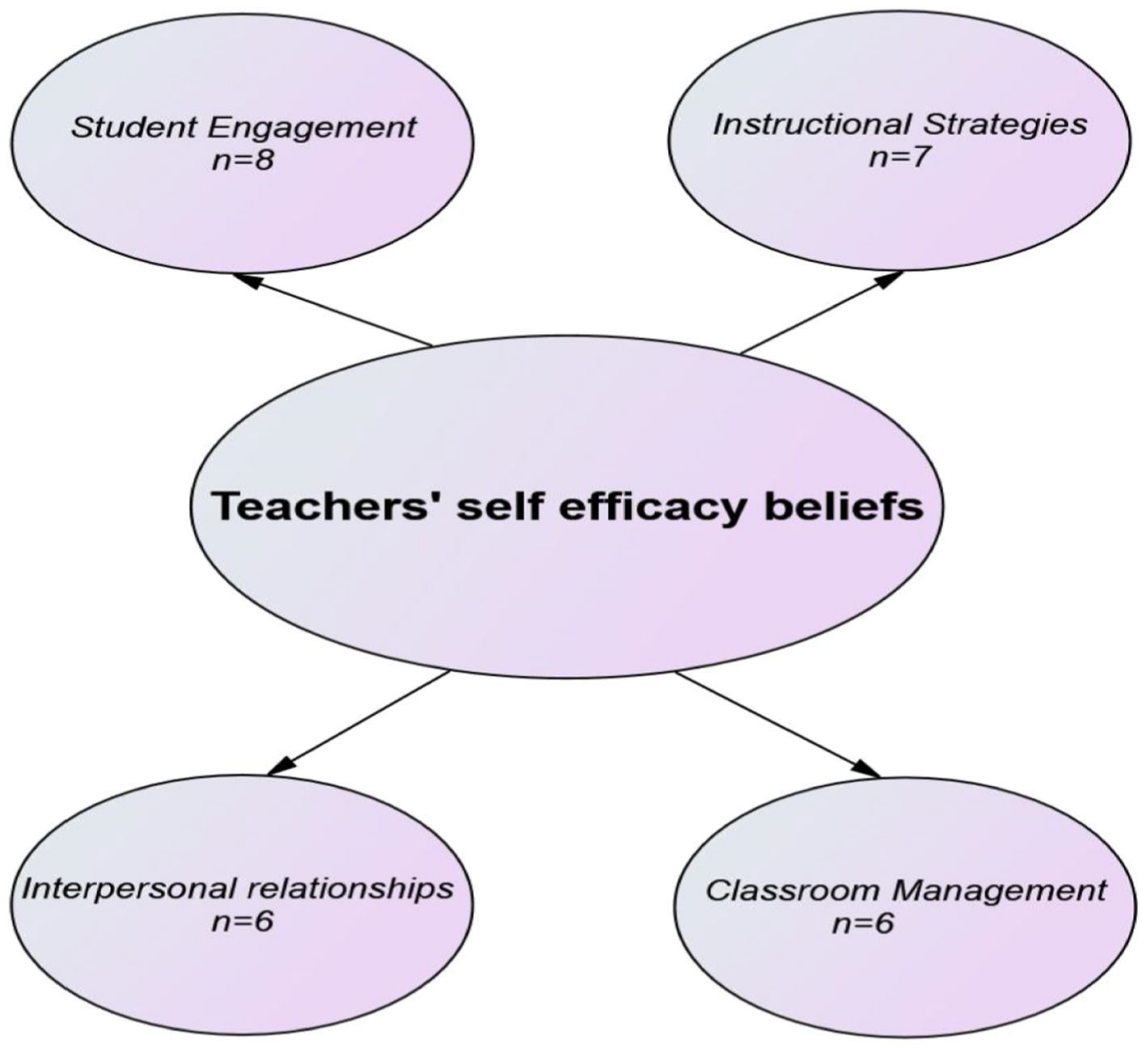
Teachers’ self-efficacy beliefs.

As for the qualitative findings, based on the inductive content analysis of the qualitative data obtained from the study, they were collected in four main themes. Accordingly, the themes identified as “Student Engagement” (*n* = 8), “Instructional Strategies” (*n* = 7), “Interpersonal relationships” (*n* = 6) and “Classroom Management” (*n* = 6) were given in Figure 2.

### Student engagement

In the participant teachers’ teaching experiences, “Student Engagement” was described as one of the themes emerging in the data. Out of the 10 participants in this cohort, seven commented on the “Student Engagement” associated from various standpoints. T8 with a feeling of self-efficacy stressed that a lesson becomes very effective if the learners are involved in it and T8 with a feeling of self-confidence and happiness was skillful in realizing it:

“When all students are very engaged and talk to each other about the content, a lesson goes really well and this makes me feel really happy. I am proud of myself because I am doing something right. I can state clearly that I can involve my students in my course.” (T8)

Another participant who stated that he can cope with student engagement problems by identifying the source of the problem and trying a variety of techniques or materials in order to make them involved in the course:

“I can effectively engage the students in the lesson. When my students are not interested in the lesson, I talk to them and learn what the problem is. By doing so, I sometimes can find solutions for them to be involved in the course. Additionally, I encourage them and try a variety of techniques or materials considering their interests to achieve student engagement.” (T3)

T5 who also stated that he can make learners involved in the course:

“I try very hard and manage to engage the students successfully by providing them enough information and supporting them to understand the topic as well as choosing something interesting enough to get their attention.” (T5)

As shown in the extracts above, participants emphasize that they are self-efficient for student engagement that facilitates learning and teaching experiences. At the same time, they know how to cope with student engagement problems in the course.

### Instructional strategies

The second theme described in participant teachers’ data was that of “Instructional Strategies.” T4 was self-efficient about the use of instructional strategies based on the awareness of students’ individual characteristics and stressed:

“I believe that I’m really successful in using teaching strategies. I take into account my students’ levels, interests, and expectations. I try to use various teaching strategies.” (T4)

Some participants stated that they are skillful in the use of a variety of instructional strategies and assignments as well as individualized instruction focusing on the needs of the individual student. Some common responses were:

“I am good at using different teaching methods, which helps me conduct courses effectively, and I can say that I always manage to do it.” (T1)

“I adapt to various teaching strategies in every kind of classroom to make the lesson more efficient. The mixed-ability classes, where you teach the same curriculum using several approaches, have so far been the most affected. Namely, different kinds of assignments, instructional methods, and approaches to each student work well.” (T6)

“… to be honest, I know exactly how to apply teaching techniques. I put myself in the place of my students and strive to answer questions myself. So, I learn how to use these techniques more clearly.” (T9)

As shown in the quotations above, participants understated that they were self-efficient for instructional strategies and they are quite aware of the fact that individualized instruction is of great importance in order to achieve efficacy for instructional Strategies.

### Interpersonal relationships

The third theme identified in participant teachers’ data was that of “Interpersonal relationships.”

T10 with a strong sense of self-efficacy about great relationship with the students conveyed:

“I think I am a teacher whom my students are not afraid of and easily ask questions to I mean I am approachable. They are comfortable to make requests. I can communicate well with my students.” (T10)

In a similar vein, some participants were of the opinion that they were self-efficient about interpersonal relationships with their students and teacher-student relationships were of great importance with regard to instruction process. Some common responses were:

“I share a lot with my students, and they aren’t afraid of me. I mean my course is not like a course for them. I turn it into an entertaining course. I am a teacher who is immensely helpful to my students.” (T3)

“Before I begin teaching, I firstly establish a supportive but friendly rapport with my students. It works effectively and all of the learners are attentive and focused on completing their classwork.” (T2)

“First of all, I can say that I have a love bond with my students. I mean I treat them like my own children. I take a close interest in their problems. I mean this way I can personally establish a bond with my students and reach them.” (T7)

“I’m close to my students as a teacher. I believe it’s important to maintain a boundary with students in terms of teacher-student relationships. I mean I’m this kind of a teacher who can have a strong communication with his/her students and help them.” (T5)

As demonstrated in the excerpts above, participant teachers with a strong sense of self-efficacy about interpersonal relationships were of the belief that developing positive relationships with students made teaching and learning experiences easier. Additionally, they thought that this could encourage them to participate actively and to concentrate during class.

### Classroom management

The fourth theme that emerged as a result of the analysis of participant teachers’ data was identified as “Classroom Management.” T3 conveyed that the objective was to set the tone of the class straight away by introducing in-class rules and policies to adhere to.

“I push my students to do their work in class. We establish the rules for our democratic class on the first day. My students behave nicely and always follow the rules. They do not have an opportunity to misbehave in the class.” (T3)

Another participant teacher believed that it was necessary to maintain a relaxed atmosphere in class to feel at ease.

“It’s important to have some humor and play quick games in between lessons to make the learning environment enjoyable. I can do it easily.” (T8)

T9 with a sense of self-efficacy about classroom management conveyed:

“I am aware of every student’s level and needs. I can manage my classroom considerably better in terms of behaviour and classroom management.” (T9)

T1 also emphasizes that she overcame with classroom management in class by keeping students engaged in learning and acquiring knowledge.

“I learned from my previous teaching experiences that busy learners are more manageable. When students are very cooperative, and they participate in class discussions, all of them are easy to manage in terms of classroom behaviour.” (T5)

On the basis of the aforementioned data, it can be concluded that participant teachers were aware of when and how to utilize various forms of class control and they were self-efficacy about classroom management.

## Results and discussion

Personal judgments of an individual’s capacity to deal with various realities in life are so significant that they have a higher potential to affect their behavior than any other beliefs or thoughts (Bandura, 1986). This view maintains the importance of self-efficacy beliefs for teachers. In light of this, the current study investigated the teachers’ self-efficacy views. Based on an examination of the mixed data, it was determined that teachers had optimistic views of their own self-efficacy beliefs.

Taking into account the qualitative and quantitative results of the present study, we found that teachers’ self-efficacy levels were high, and they felt self-efficient in their teaching, which was supported by similar studies (Turcan, 2011; Eker, 2014; Buluç and Demir, 2015). Considering that self-efficacy belief may affect the teacher’s effort and feedback behavior during teaching, high self-efficacy levels of teachers can be stated to increase the quality of education (Gibson and Dembo, 1984). Furthermore, it can be deduced from the comments made by the participant teachers that managing challenging students, interacting socially with them, teaching achievement, and skill development on the teaching profession are the main domains for which the participants may have varying expectations about their own self-efficacy. In reality, these important areas seem to be essential for effective schooling.

The study also uncovered that teacher self-efficacy does not significantly differentiate by gender. Similar findings were also found by some researchers (Tschannen-Moran and Woolfolk Hoy, 2002; Akbaş and Çelikkaleli, 2006; Azar, 2010; Fettahlıoğlu et al., 2011; Duban and Gökçakan, 2012; Ekinci et al., 2014) wheras there exist studies with different findings revealing that teacher self-efficacy significantly differentiates in favor of female teachers (Yalçın, 2011; Kurt and Ekici, 2012; Arpacı and Birhanlı, 2013) and in favor of male teachers (Morgil et al., 2004). The study revealed that there is no statistically significant difference between total scores of teachers’ self-efficacy perceptions in terms of the school level. In other words, it can be said that teachers’ self-efficacy perceptions are similar in terms of the school level.

When we examined the qualitative and quantitative results of the present study simultaneously, it was revealed that the top 10 participants with the highest level of self-efficacy were composed of the teachers who had a seniority of over 21 years and had graduate education (Master/PhD). It can be thought in the context of the current study that the perception of “self-efficacy” increases with the increase in seniority and as the level of education increases, the knowledge in the field deepens and affects teachers’ self-efficacy beliefs positively. As for the participant teachers’ seniority, the current study revealed that teachers’ self-efficacy significantly differentiated by seniority and that the self-efficacy beliefs of teachers with a seniority of 1–10 years were significantly lower than those of teachers with seniority of 11–20 years and over 21 years. When the relevant literature is investigated, it is observed that there are similarly significant relationships between the seniority of teachers and their self-efficacy beliefs (Glickman and Tamashiro, 1982; Dembo and Gibson, 1985; Evans and Tribble, 1986; Rubeck and Enochs, 1991; Soodak and Podell, 1996; Lamorey and Wilcox, 2005; Tschannen-Moran and Hoy, 2007; Aydın et al., 2016). Tschannen-Moran and Woolfolk Hoy (2002) informed that experienced teachers find opportunities to develop effective instructional strategies and classroom management skills over time (p. 6). According to Bandura (1986, 1997), the most powerful sources of efficacy beliefs are previous or mastery experiences, and empirical research (e.g., Palmer, 2006; Menon, 2020) support this. In fact, the participants’ current high level of self-efficacy views may have been influenced by their prior experiences. The studies of Campbell (1996), Daugherty (2005), and Tschannen-Moran and Woolfolk Hoy (2002) reached the conclusion that the perception of “self-efficacy” increases with the increase in seniority whereas in some studies (Celep, 2002; Chacon, 2005), no difference was found between seniority and self-efficacy perception. The present result obtained from our study may support that younger teachers may feel inadequate due to their inexperience or negative experiences compared to teachers of other ages. It can be said that this existing situation negatively affects self-efficacy belief. As for the participant teachers’ the level of education, the study also uncovered that self-efficacy levels of the teachers displayed a significant difference in support of the teachers who had graduate education (Master/PhD). In a similar direction, Yılmaz and Çokluk-Bökeoğlu (2008) also found the self-efficacy levels of primary school teachers with graduate education as being higher than the teachers who had undergraduate education. With regard to the finding obtained from our study, it can be thought that as the level of education increases, the knowledge in the field deepens and affects teachers’ self-efficacy beliefs positively whereas there exist studies with different findings showing that the increase of pre-service teachers’ self-efficacy regards only the first years of university (Zach et al., 2012). In the present study, the top 10 participants with the highest level of self-efficacy were revealed to have teaching experiences identified as the themes emerging in the data such as “Student Engagement,” “Instructional Strategies,” “Interpersonal relationships,” and “Classroom Management.” Firstly, the participants emphasized that they were self-efficient for student engagement that facilitates learning and teaching experiences. This finding consistent with the study by Narayan and Lamp (2010) that students’ being actively involved in activities in the course can aid in increasing teachers’ self-efficacy. Additionally, as Tschannen-Moran and Hoy (2007) claim, the discovery of techniques that may enhance this skill leads to the steady development of student engagement, which is a more complex work for teachers. Secondly, the participants understated that they were self-efficient for instructional strategies. In fact, the present finding provides evidence for Bandura’s self-efficacy theory, Gagne’s theory of instruction, and their viewpoint on teacher self-efficacy in the teaching profession, which holds that teaching strategies have a significant influence on teachers’ self-efficacy views. Helping teachers better implement a variety of teaching strategies may thereby increase their confidence and self-efficacy as educators. Teachers’ confidence in their ability to successfully teach their classes may rise when their usage of instructional strategies is enhanced. Thirdly, the participants with a strong sense of self-efficacy about interpersonal relationships believed that developing positive relationships with students made teaching and learning experiences easier. Finally, the participants in the study felt self-efficient about classroom management and knew when and how to utilize various forms of class control.

In this study, there are some restrictions to be stated. To begin with, this study only included teachers who were employed at seven different schools in a Turkish city as participants. At the same time, the top 10 participants with the highest level of self-efficacy were chosen for the qualitative dimension of the study. Therefore, it is difficult to generalize to all Turkey. To gain a wider view on teachers’ self-efficacy beliefs, a national survey may be realized. Additionally, there were a limited number of participants for both quantitative and qualitative data. To get a more accurate picture of the issue at hand, future studies with a large number of participants can be conducted.

In addition, the majority of this study’s data came from participants’ self-reported assessments of their self-efficacy beliefs. Additional research can use a proficiency test to gauge the actual level of teacher proficiency and compare the outcomes.

Future research may benefit from the conclusions of this study. As suggested in the literature, teachers’ efficacy belief is a complex concept that differs depending on the tasks and teaching environments. To better understand teachers’ ideas about their own self-efficacy in various circumstances, more research must be done. Besides, more research that focuses on the viewpoint of teachers can be done to ascertain how teachers’ self-efficacy views affect their instruction. To investigate teachers’ levels of self-efficacy and how they perform as teachers, in-class observations may be used as an additional source of data.

## Interview protocol

1. I would like to ask you to think broadly about your self-efficacy as a teacher and give me about your self-efficacy experiences during teaching.
2. Tell me about some of the most prominent self-efficacy components in teaching that you experience when you are teaching and when you are preparing to teach.
3. What gives you the confidence that you can manage your class well? Describe your self-efficacy in regards to classroom management.
4. What gives you the confidence that you can engage your students? Describe your self-efficacy in regards to student engagement.
5. What gives you the confidence that you can use the appropriate instructional strategies? Describe your self-efficacy in regards to in regards to instructional strategies.
6. What gives you the confidence that you can build strong relationships with your students? Describe your self-efficacy in regards to the relationship with your students.

## Data availability statement

The raw data supporting the conclusions of this article will be made available by the authors, without undue reservation.

## Ethics statement

The studies involving human participants were reviewed and approved by the Ministry of National Education of Türkiye (dated 09.11.2021, approval number 19632675). Written informed consent to participate in this study was provided by the participants’ legal guardian/next of kin.

## Author contributions

All authors listed have made a substantial, direct, and intellectual contribution to the work and approved it for publication.

## Conflict of interest

The authors declare that the research was conducted in the absence of any commercial or financial relationships that could be construed as a potential conflict of interest.

## Publisher’s note

All claims expressed in this article are solely those of the authors and do not necessarily represent those of their affiliated organizations, or those of the publisher, the editors and the reviewers. Any product that may be evaluated in this article, or claim that may be made by its manufacturer, is not guaranteed or endorsed by the publisher.
